# New metallo-β-lactamase: Is its name after New Delhi helpful or harmful?

**Published:** 2011-04

**Authors:** Manoj Jain, T.J. John

**Affiliations:** Rollins School of Public Health at Emory University, 1518 Clifton Road Atlanta, Georgia 30322, USA mkjain@aol.com

Sir,

We read with great interest the editorial on the new metallo-β-lactamase^1^. It gives a clear picture of β-lactamases in general and the new one named NDM-1 in particular. It is also an alert on “the disorganized state of antibiotic therapy in India”^1^. While addressing the lack of evidence for New Delhi as the place of origin of the plasmid encoding the enzyme, the author states that “our national sentiments are tickled” and that its naming (after New Delhi) “is not really objectionable but questionable”^1^.

While several metallo-β-lactamases have been named after cities where they originated or were detected first without deleterious effects, the naming of the new one with our nation’s capital has had much negative impact^2^,^3^. It was not detected in New Delhi, and there is insufficient evidence of its place of origin^1^,^2^. The name was misused in one paper to warn people to avoid India as a destination for surgical treatment as if the problem was predominantly and almost exclusively in India^4^. The editor of that journal has even tendered a public apology for publishing it^5^. The plasmid in question is globally widespread as are many others carrying other β-lactamases^6^. There is a public appeal on the electronic web, requesting the removal of New Delhi from the name, already signed by over 5000 individuals^3^.

The objection to naming the plasmid after India’s capital actually arose after its misuse for discouraging medical tourism in India which is an economically growing enterprise^7^. Thus, its naming has become objectionable in addition to being questionable. We propose that the name be changed, although such a step is unprecedented. However, there are already over 30 β-lactamases carrying alternate names^8^.

We also propose that hereafter names of places or persons be avoided for newly discovered biological agents.

Has the name helped in increasing public perception of the “disorganized state of antibiotic therapy” in India? The answer is no, but on the other hand the media and even government officials went into a defensive mode, thus missing a good opportunity to publicly discuss the need for organization and discipline of antibiotic availability, choice and clinical use so as to reduce the spread of existing antibiotic-resistant microbes and to prevent others from developing. We know that D. Ragunath is a leader in the area of antimicrobial resistance. We urge him, the Indian Council for Medical Research, the Departments of Health Research and Health & Family welfare and other concerned agencies to take the combat against antibiotic misuse on a ‘war footing’ with enforceable regulations and legislations.

## References

[1] Ragunath D (2010). New metallo-β lactamase NDM-1. *Indian J Med Res*.

[2] Yong D, Toleman MA, Giske CG, Cho HS, Sundman K, Lee K (2009). Characterization of a new metallo-beta-lactamase gene, bla (NDM-1), and a novel erythromycin esterase gene carried on a unique genetic structure in *Klebsiella pneumoniae* sequence type 14 from India. *Antimicrob Agents Chemother*.

[3] http://www.changesuperbugname.com.

[4] Kumarasamy KK, Toleman MA, Walsh TR, Bagaria J, Butt F, Balakrishnan R (2010). Emergence of a new antibiotic reistance mechanism in India, Pakistan, and the UK: a molecular, biological, and epidemiological study. *Lancet Infect Dis*.

[5] Kounteya S Lancet says sorry for ’Delhi bug’.

[6] Koh TH, Khoo CT, Wijaya L, Leong HN, Lo YL, Lim LC (2010). Global spread of New Delhi metallo-beta-lactamase-1. *Lancet Infect Dis*.

[7] Shetty P (2010). Medical tourism booms in India, but at what cost?. *Lancet*.

[8] Jacoby G (2006). Mini review.Beta lactamase nomenclature. *Antimicrob Agents Chemother*.

